# Safety and Efficacy of Humanized Versus Murinized CD19 and CD22 CAR T-Cell Cocktail Therapy for Refractory/Relapsed B-Cell Lymphoma

**DOI:** 10.3390/cells11244085

**Published:** 2022-12-16

**Authors:** Lefu Huang, Jingjing Li, Junfang Yang, Xian Zhang, Min Zhang, Jiujiang He, Gailing Zhang, Wenqian Li, Hui Wang, Jianqiang Li, Peihua Lu

**Affiliations:** 1 Department of Basic Medical Sciences, Tsinghua University School of Medicine, Beijing 100084, China; 2 Lu Daopei Institute of Hematology, Beijing 100176, China; 3 Hebei Yanda Lu Daopei Hospital, Langfang 065201, China; 4 Hebei Senlang Biotechnology Co., Ltd., Shijiazhuang 050000, China; 5 Key Laboratory of Immune Mechanism and Intervention on Serious Disease in Hebei Province, Department of Immunology, Hebei Medical University, Shijiazhuang 050000, China

**Keywords:** CAR-T, aggressive B-cell lymphoma, cocktail therapy, humanized, murinized

## Abstract

CD19 chimeric antigen receptor T-cell (CAR-T) therapy is efficacious for refractory/relapsed (R/R) B-cell hematological malignancies, yet relapse due to CD19 antigen escape remains a challenge. Our trial explored simultaneous targeting of multiple B-cell antigens as a therapeutic approach that may reduce the risk of relapse. We tested the safety and efficacy of CAR19/22 T-cell cocktail therapy including murinized and humanized products among patients with R/R aggressive B-cell lymphoma. In the group that received the humanized product, 11/12 (91.7%) patients achieved an objective response, including 9/12 (75%) complete responses (CRs) by day 28. The overall response rate and CR rate in the murinized group was 92.9% (13/14) and 42.9% (6/14), respectively. Nine of 12 (75%) patients in the humanized group maintained CR at month 3 following infusion, compared to 5/14 patients (35.7%) in the murinized group. Progression-free survival (PFS) was more favorable in the humanized compared to the murinized group. Most patients had mild cytokine release syndrome (CRS) (grade 1–2) in both groups. This study demonstrates that CAR19/22 T-cell cocktail therapy is safe and effective for R/R B-cell lymphoma and that patients treated with a humanized CAR-T exhibited better efficacy compared to patients treated with a murinized CAR-T therapy.

## 1. Introduction

CD19 chimeric antigen receptor T-cell (CAR-T) therapy has resulted in an impressive, high clinical response rate among patients with refractory/relapsed (R/R) B-cell hematological malignancies, yielding about a 90% complete response (CR) rate in B-cell acute lymphocytic leukemia (B-ALL) and about a 50% CR rate in B-cell non-Hodgkin’s lymphoma (B-NHL) [1,2,3]. Despite these remarkable response rates, approximately 30% to 50% of patients treated with single-target CD19 CAR-T cells relapse within 1 year, including with CD19^+^ and CD19^−^ relapse [3,4,5], and particularly among patients characterized by high-risk factors such as double-hit lymphoma, high tumor burden, and presence of a TP53 mutation [4,5,6].

Biopsies from patients who relapse following CD19 CAR-T cell therapy reveal that approximately 20% to 30% of patients have CD19^−^ relapse [5,6,7]. CD19^−^ relapse is related to the down regulation or loss of the CD19 target antigen on cancer cells and the emergence of CD19^−^ clones. The common mechanisms of resistance include changes in the target genes (such as alternative splicing and acquired missense mutations), tumor cell phenotype transformation and clone selection, and cytoplasmic exchange (tumor cells’ abnormal transfection) [5,6,7,8]. Simultaneous targeting of multiple B-cell antigens has been proposed as a therapeutic strategy to reduce the risk of relapse mediated by antigen loss. Similar to CD19, CD22 is also expressed on the surface of most B-cell malignancies and is also an inhibitory co-receptor [9,10]. Several studies have demonstrated that CD22^+^ CAR-T cells induce about an 80% CR rate and have equal potency in CD19^+^ and CD19^−^ B-ALL [11]. Therefore, combination therapy targeting both CD19 and CD22 likely targets heterogeneous cell subpopulations in patients with primary and recurrent B-cell malignancies, with a low probability of simultaneous loss of both antigens that could lead to resistance. Correspondingly, these CAR-T cells retain the ability to eliminate tumor cells even if the loss of one of the target molecules occurs, which greatly inhibits the recurrence of malignant cells.

CD19^+^ relapse is generally associated with insufficient persistence and poor potency. The design of CAR-T, including the costimulatory domain and the source of the CAR single-chain variable fragment (scFv), is the main factor affecting its persistence and potency [12]. Most clinically registered CARs contain single-chain variable fragment (scFv) domains derived from murinized monoclonal antibodies. To date, there are five FDA-approved CAR T-cell products based on murine scFvs to redirect their specificity against target antigens, including four targeting CD19 and one targeting B-cell maturation antigen (BCMA) [12,13,14,15]. The immunogenicity of CAR-T causes an anti-CAR immune response, which in turn rejects, deletes, and clears CAR T-cells. The immunogenicity of CAR-T is also an important factor affecting the long persistence of CAR-T cells in vivo. Therefore, strategies such as humanization and replacement of ligand receptor domains to reduce the immunogenicity of CAR are important considerations for CAR-T cell design. Particularly, some preclinical studies have suggested that CARs with fully humanized single-chain variable fragments (scFvs) might evade the potential host anti-CAR immune response and maintain antitumor activity. However, there are relatively few clinical trials comparing the differences between humanized and murinized CAR T-cell therapy in recurrent B-cell NHL.

We previously described a dual-targeted therapy composed of CD19^+^ and CD22^+^ CAR-T cell cocktail, which has yielded a 93.3% CR rate in R/R B-cell acute lymphoblastic leukemia (B-ALL) and could reduce the risk of CD19^−^ relapse reported previously [16]. Based on these promising outcomes, here we developed a humanized CAR-T with a fully human scFv based on the backbone of a murine-based CAR and designed a phase I/II clinical trial (NCT05206071) to compare the safety and efficacy between humanized and murinized CAR19/22 T-cell cocktail therapy for R/R aggressive B-cell NHL.

## 2. Methods

### 2.1. Construct Design and Generation of CD19 and CD22 CAR-T Cell Cocktail

The CD19 and CD22 CAR T-cell cocktail was manufactured in a cGMP facility. Peripheral blood mononuclear cells (PBMCs) were first collected and CD3^+^ T cells were separated using CD3 microbeads. The lentiviral vector contained constructs of CD19 or CD22-targeting CARs that infect auto-T-cells separately (Supplementary Figure S1). The murinized CD19 CAR construct is composed of a scFv sequence derived from a monoclonal antibody (FMC63) [17], a CD8a hinge, transmembrane domain, 4-1BB costimulatory domain, CD3ζ activation domain, and an additional truncated EGFR sequence, providing a cell surface marker for in vivo tracking of adoptively transferred T cells using flow cytometry [18]. The humanized CAR T-cells were developed by replacing the entire mouse-derived scFv with fully human scFvs based on the backbone of a murine-based CAR to weaken their immunogenicity. The humanized CD19 CAR T-cells were developed by replacing the entire mouse-derived scFv with a fully human scFv [19] based on the backbone of a murine-based CAR to weaken their immunogenicity. The humanized CD22 CAR is composed of a fully human anti-CD22 scFv sequence (m971) [20], a CD8a hinge, transmembrane domain, 4-1BB, and CD3ζ activation domains, and an additional anti-PDL1 scFv. The CD19 and CD22 CAR T-cells were cultured separately for 12–14 days until a sufficient number of cells were mixed for infusion.

### 2.2. Successful CD19 and CD22 CAR-T Manufacture for Clinical Application

For clinical application, we generated the CD19 and CD22 CAR-T product from patients’ blood. When applicable, each patient’s PBMC and cerebrospinal fluid (CSF) were collected before and after infusion to assess the persistence of CAR T-cells. Real-time quantitative PCR using primers with specificity for the scFv of CD19 CAR and scFv of the CD22 CAR was used to detect the copies of CAR19 and CAR22 T-cells transgenes, respectively, on days 0, 4, 7, 14, 21, and 28 after infusion. The percentages of CD19 and CD22 CAR T-cells in peripheral blood (PB) were detected at multiple time points by flow cytometry (FCM) by staining with an antibody against EGFR and a CD22-Fc fusion protein, respectively. Lymphocyte subsets in PB were also observed simultaneously.

### 2.3. Study Design and Procedures

A phase I/II clinical trial was conducted to evaluate the safety and efficacy of humanized and murinized CAR19/22 T-cell cocktail therapy for R/R aggressive B-cell NHL. Patients between the ages of 3 and 75 years of age who were refractory or relapsed after their prior treatments were eligible. Detailed eligibility criteria are provided in the Supplementary Methods.

After enrollment, PBMCs were collected from whole blood patient samples. All enrolled patients received fludarabine 30 mg/m^2^/d and cyclophosphamide 250 mg/m^2^/d for 3 consecutive days (day-5 to day-3) for lymphodepletion chemotherapy prior to cocktail CAR-T cells infusion. Bridging therapy was allowed between PBMC collection and lymphodepletion. CAR19 T-cells with a median dose of 2 × 10^6^/kg (1–3 × 10^6^/kg) and CAR22 T-cells with a median dose of 1 × 10^6^/kg (0.5–3 × 10^6^/kg) were mixed and infused together on day 0.

Positron emission tomography-computed tomography (PET-CT) was performed as the diagnostic imaging to evaluate efficacy before infusion, on day 28, and month 3. Bone marrow evaluation was performed one month before and after CAR-T infusion to assess whether bone marrow was infiltrated. All patients were followed up until they were lost to follow-up, withdrew consent, or died.

### 2.4. Endpoints and Assessments

The primary endpoint of the trial was to evaluate the short-term efficacy and safety of CD19/CD22 CAR-T cocktail therapy. Efficacy assessment was conducted on day 28 and month 3 post CD19/CD22 CAR-T cocktail cell infusion according to the 2014 Lugano classification or the International Working Group Response Criteria for Malignant Lymphoma using PET/CT images evaluated by two expert nuclear medicine physicians. The objective response rate (ORR) was defined as the percentage of patients who achieved partial response (PR) or CR.

Cytokine release syndrome (CRS) and immune effector cell-associated neurotoxicity syndrome (ICANS) were graded according to the ASTCT Consensus. The CRS was judged as severe if it was grade 3 or above [21]. Severe neurotoxic effects were defined as a seizure of any grade or a toxic effect of grade 3 or above. Other adverse events were graded according to the National Cancer Institute Common Terminology Criteria for Adverse Events (CTCAE) version 5.0 [22,23]. Cytokines in PB, including interleukin (IL)-2, IL-6, IL-2R, and tumor necrosis factor-a (TNF-a), transforming growth factor-β1 (TGF-β1), interferon gamma (IFN-γ), and monocyte chemotactic protein 1(MCP-1) were detected at multiple time points by enzyme-linked immunosorbent assay to help explore the severity and cause of adverse events.

The secondary endpoint was to evaluate the progression-free survival (PFS) and overall survival (OS) after CD19/CD22 CAR-T cocktail therapy. The PFS was defined as the time from CAR-T infusion until relapse, progression, or death from any cause. OS was defined as the time from CAR-T infusion until death of any cause. The data cutoff date was 31 May 2022.

### 2.5. Ethics

The study protocol was approved by the Regional Ethical Review Board of Hebei Yanda Lu Daopei Hospital. Patients were treated according to the Declaration of Helsinki’s ethical principles for medical research involving human subjects. Written informed consent was obtained from all patients or their guardians prior to study enrollment.

### 2.6. Statistical Analysis

Continuous variables were expressed as mean ± SD (standard deviation) and compared using a two-tailed unpaired Student’s t test. Categorical variable analysis was performed using the Clopper–Pearson 95% confidence interval (CI) and Fisher’s exact test. The independent risk factors found to be significantly related to PFS and OS were estimated using the Kaplan–Meier method and compared with the log-rank test. Statistical analyses were performed by using SPSS version 22 (IBM SPSS Statistics, IBM Corporation, Armonk, NY, USA) and GraphPad Prism 8 (GraphPad Software, La Jolla, CA, USA). A value of *p* < 0.05 was considered significant in all analyses.

## 3. Results

### 3.1. Patient Characteristics

**Figure 1:**
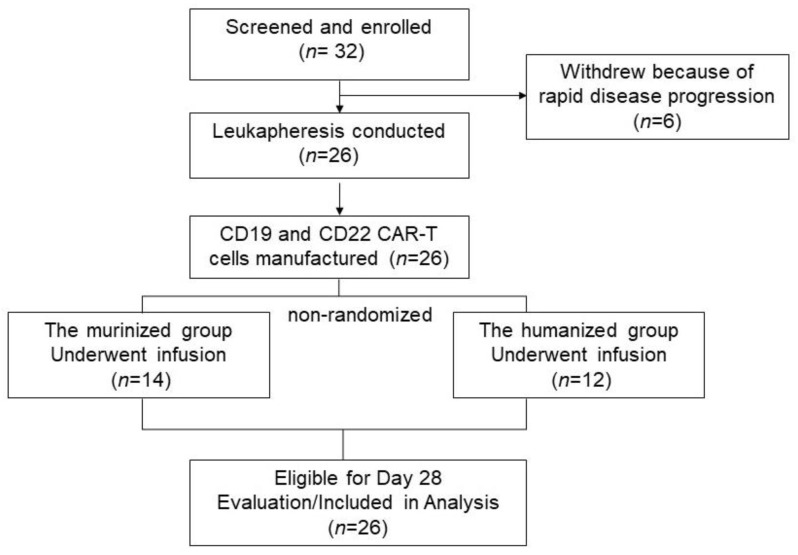
Consort diagram of patient flow. 32 patients were enrolled in the study, 6 patients withdrew because of disease progression, and 26 patients successfully underwent leukapheresis and CD19/22 cocktail CAR-T cell infusion. All 26 patients were available for day 28 evaluation post CAR-T cells infusion. CAR-T: chimeric antigen receptor-T cell.

From July 2020 to December 2021, 32 patients with R/R B-cell NHL were initially screened and enrolled in this study. Six patients withdrew due to rapid disease progression and were excluded from the study analysis (Figure 1). A total of 26 patients (14 patients received the murinized CAR19/22 cocktail infusion and 12 received the humanized CAR-T) were eligible for the efficacy and safety evaluation on day 28. Patient baseline characteristics are detailed in Table 1 and summarized in Supplementary Table S1. The median age was 50.5 years (range: 29–66 years old) and 46 years (range: 4–75 years old) in the murinized and humanized groups, respectively. All features between the two groups were as balanced as possible, except for four patients (Pt09, Pt10, Pt13, and Pt17) who had relapsed after prior murine CD19 CAR-T cell therapy and who were enrolled in the humanized cohort directly. There were two Burkitt lymphoma and 10 diffuse large B-cell lymphoma patients in the murinized and humanized groups, respectively. The majority of patients were in second or greater relapse and had received a median of 3 lines of therapy (range: 2–5 lines) prior to enrollment in the current trial. Additionally, three patients were diagnosed with double-hit lymphoma in the murinized group, carrying MYC and BCL2 or BCL6 translocations simultaneously, compared to four patients in the humanized group. Frequency of TP53 mutation was also similar between the two groups—six cases in the murinized group and five in the humanized group. Additionally, one patient in each group had a prior relapse following autologous hematopoietic stem cell transplantation (HSCT).

### 3.2. Manufacturing and Infusion of CAR19 and CAR22 T-Cells

The CD19 and CD22 CAR T-cells were successfully manufactured and effectively transduced for all 26 patients. The incubation time was approximately two weeks. The median transduction efficiency of CAR19 and CAR22 T-cells was 31% (range: 12.3–52%) and 27.1% (range: 14–80.1%) in the murinized group, respectively, and 36.2% (range: 11.1–47.2%) and 10.55% (range: 3.21–72.8%) in the humanized group, respectively. Additionally, the mean fluorescence intensities (MFI) of CAR expression of CAR19 and CAR22 T-cells were also similar in both groups (Supplementary Figure S2).

In general, CD19 and CD22 CAR T-cells were mixed into cocktails in 2 divided doses and then infused together on day 0. Fourteen patients received the murinized CAR19 and CD22 CAR T-cells at a median dose of 2 × 10^6^/kg (range: 1–3 × 10^6^/kg) and 1 × 10^6^/kg (range: 1–3 × 10^6^/kg), respectively, while 12 patients received the humanized CD19 and CD22 CAR T-cells at a median dose of 2 × 10^6^/kg (1–3 × 10^6^/kg) and 0.5 × 10^6^/kg (0.5–3 × 10^6^/kg), respectively. The doses of the infused CAR T-cells were related to the tumor burden and the specific condition of each patient before infusion (Supplementary Table S1).

### 3.3. Patients Who Received Humanized CAR T-Cells Achieved a Higher CR Than Those Treated with the Murinized CAR-T, Even among TP53 Mutation-Positive Patients

**Figure 2:**
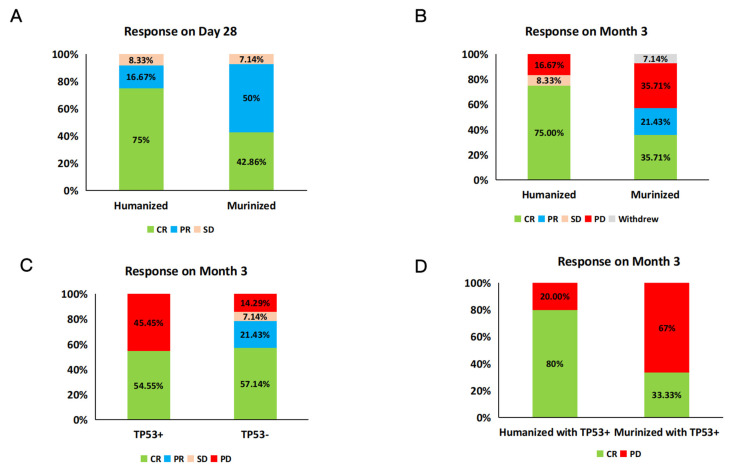
Response comparison between the murinized and humanized groups. Shown are the clinical outcomes of the murinized group and humanized group on day 28 (**A**) and month 3 (**B**). (**A**) On day 28, the humanized group had a higher CR rate than the murinized group (75% vs. 42.9%). (**B**) At month 3, the percentage of patients achieving CR in the murinized group and humanized group were 35.7% and 75.0%, respectively. Pt03 withdrew from the murinized group. (**C**) The various response at month 3 between the patients with TP53+ and TP53−. The patients with TP53- had a better ORR than the patients with TP53+. (**D**) The response comparison between the patients with TP53+ in the humanized group and the murinized group at month 3. The CR rate of the patients with TP53+ in the humanized group and the murinized group were 80% and 33.33%, respectively. CR: complete response; PR: partial response; SD: stable disease; PD: progressive disease. ORR: objective response rate.

Our primary efficacy analysis showed that 91.7% of patients (11/12) achieved objective response including 75% (9/12) complete responses (CRs) and 16.7% (2/12) partial responses (PRs) in the humanized group on day 28 following infusion (Figure 2A). This is compared with 42.9% of patients (6/14) who obtained a CR and 50% of patients (7/14) who obtained a PR in the murinized group on day 28 after infusion. During the month 3 evaluation, 75% of patients (9/12) were still in their initial CR without progressive disease (PD) in the humanized group, while the overall response rate (ORR) and CR rate in the murinized group were 57.1% (8/14) and 35.7% (5/14) (Figure 2B), respectively. One patient (Pt03) withdrew from the study. Of the 4 patients who relapsed after prior murine, single-target CD19 CAR T-cell therapy, 2 patients (Pt09, Pt17) achieved and maintained a CR to data, 1 patient (Pt13) had a PR on day 28 but progressed in less than three months, and another patient (Pt10) had no response (NR) to CAR19/22 T-cell cocktail therapy (Supplementary Table S1). Additionally, all 4 patients (Pt01, Pt08, Pt18, and Pt25) with Burkitt lymphoma in both groups achieved a CR on day 28, and only 1 of these patients (Pt01) progressed within 3 months and died due to disease progression. The remaining 3 Burkitt’s lymphoma patients maintained remission to date, including one who continued long-term remission after subsequently bridging to allogeneic hematopoietic stem cell transplantation (allo-HSCT).

Of the 26 patients enrolled, patients without a TP53 mutation (*n* = 15) had a higher response rate than patients with a TP53 mutation (*n* = 11) at month 3 (78.6% vs. 54.56, *p* = 0.2011) (Figure 2C). Among the TP53 mutation-positive patients, those who received a humanized cell infusion had a higher response rate than those who received murinized CAR-T (80% vs. 33%, *p* = 0.1217) (Figure 2D), and their response rates were similar to TP53 mutation-negative patients (80.0% vs. 78.6%).

### 3.4. Patients in the Humanized Group Had a Longer PFS Than Those in the Murinized Group

**Figure 4:**
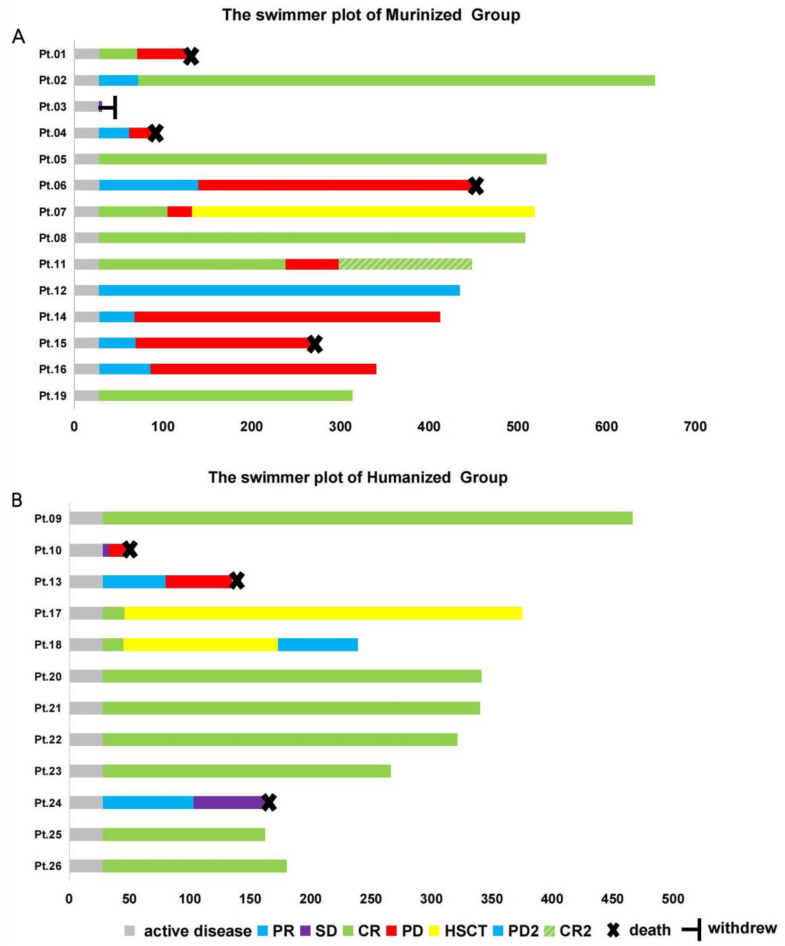
Clinical outcomes of the murinized and humanized group patients. The clinical outcomes post-murinization (**A**) and humanization (**B**). CD19/22 cocktail CAR-T infusions are shown in swimmer plots. Responses were confirmed and assessed according to the 2014 Lugano classification or the International Working Group Response Criteria for Malignant Lymphoma using PET/CT images evaluated by two expert nuclear medicine physicians. (**A**) Median follow-up time of the murinized group was 423 days (range: 31–654 days). Pt03 withdrew on day 31 due to a lack of response to CAR-T. Pt07 achieved CR on day 28, but relapsed on day 77, and later achieved CR again with radiotherapy combined with allo-HSCT. CR2 indicates achievement of CR again after PD. (**B**) Median follow-up time of the humanized group was 252.2 days (range: 47–466 days). Seven patients (Pt09, Pt20, Pt21, Pt22, Pt23, Pt25, and Pt26) had continued remission after CAR-T therapy. Two patients (Pt17 and Pt18) bridged to allo-HSCT after CAR-T therapy, 1 patient remained in CR post-HSCT, and the other relapsed on day 173. CR: complete response; PR: partial response; SD: stable disease; PD: progressive disease; allo-HSCT: allogeneic hematopoietic stem cell transplantation.

At the cutoff date of May 30, 2022, the median follow-up time was 330.5 days (range: 31–654 days). Among the 14 patients who receiving the murinized CAR T-cells, the median PFS was 140 days. The median PFS in the humanized cohort was not yet reached, but there was no statistically significant difference between the PFS of the two cohorts due to the limitation of the sample size (*p* = 0.1954, Figure 3). There was no significant difference in OS between the two groups (*p* = 0.9029), possibly due to additional subsequent therapies patients received following our trial including zanubrutinib, which improved survival in patients with progression or non-CR response. Three patients (Pt11, Pt14, and Pt16) in the murinized group who had disease progression achieved objective response, which turned into a CR as a result of subsequent zanubrutinib therapy (Figure 4A). Similarly, three patients (Pt07, Pt17, and Pt18) achieved long-term survival by undergoing subsequent allo-HSCT, including one patient (Pt07) in the murinized group who achieved CR again after disease progression following radiotherapy combined with allo-HSCT. Two patients (Pt17 and Pt18) achieved CR in the humanized group by bridging to allo-HSCT after CAR19/22 T-cell therapy (Figure 4B).

**Figure 5:**
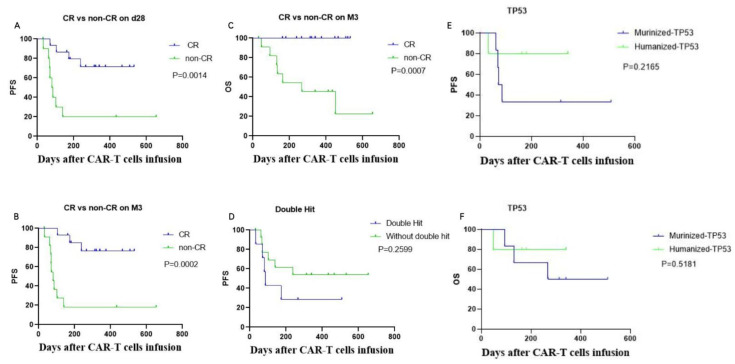
Multivariate analyses of predictive risk factors on survival outcomes. Comparison of PFS based on CR or non-CR response to CAR-T on day 28 (**A**) and at 3 months (**B**). The patients who achieved CR on day 28 and at 3 months had a better PFS than patients with non-CR (*p* = 0.0014 and *p* = 0.0002, respectively). (**C**) Comparison of OS based on CR or non-CR response to CAR-T at 3 months. There were no deaths among the patients with CR at 3 months, while the mortality of patients with non-CR was 58.3% (7/12). (**D**) Trend towards a better PFS among patients without double-hit lymphoma compared to those with double-hit lymphoma (*p* = 0.2599). (**E**,**F**) Patients with a TP53 mutation in the humanized group had a trend towards a better PFS and OS than patients with a TP53 mutation in the murinized group (*p* = 0.5181 and *p* =0.2165, respectively). OS: overall survival; PFS: progression-free survival; CR: complete response; non-CR: other responses except CR, including PR, SD, PD.

We performed multivariate analyses of predictive risk factors of survival outcomes. Achievement of CR on day 28 (HR: 0.1186, 95% CI: 0.03219–0.437) (Figure 5A) and maintenance of CR at month 3 (HR: 0.08935, 95% CI: 0.02465–0.3238) were both independent prognostic factors associated with favorable PFS (Figure 5B,C). There was also a trend towards more inferior PFS (*p* = 0.2599) for patients with double hit lymphoma versus non-double hit lymphoma (Figure 5D), although there was no significant difference between these two groups. Maintaining CR until month 3 (HR: 0.0717, 95% CI: 0.01554–0.3308) predicted longer OS outcomes. The mortality rate for patients who remained in CR until month 3 was 0%, while the mortality among non-CR patients in month 3 was 58.3% (7/12), including five patients with progressive disease (Pt01, Pt04, Pt10, Pt13, and Pt15), one patient (Pt24) with stable disease, and one patient (Pt06) with partial remission in month 3 who eventually died due to disease progression (Figure 4A). Among patients with a TP53 mutation, survival outcomes including PFS and OS in the humanized group were better than those of the murinized group. The median PFS and OS of patients with a TP53 mutation in the murinized group was 78.5 days and 388 days, respectively, while the PFS and OS of patients with a TP53 mutation in the humanized group was not yet reached (Figure 5E,F).

### 3.5. Most CRS and ICANS Were Low-Grade and Reversible

**Figure 6:**
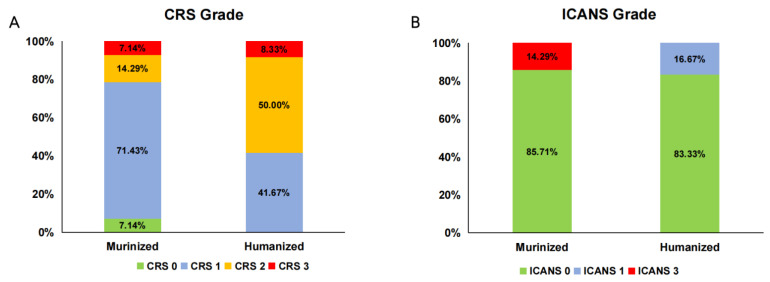
Comparison of CRS and ICANS between the murinized and humanized groups. Shown are the incidences of different grade CRSs (**A**) and ICANS (**B**) in the murinized and humanized groups. The majority of patients had mild CRS (grade 1–2) in both groups. Only 2 patients experienced grade 3 CRS, and 2 patients developed grade 3 ICANS. There was no significant difference in the incidence of CRS and ICANS between the two groups. CRS: cytokine release syndrome; ICANS: immune effector cell-associated neurotoxicity syndrome.

Although a total of 25 (96.2%) patients experienced CRS, 88.5% of cases (23/26) were mild and low grade (grade 1–2), except for 2 patients (7.7%) with bulky disease in the mediastinum, who had pleural effusion and who developed grade 3 CRS (Table 2). ICANS occurred in 15.4% of patients (4/26) within the first month after cell infusion. Among them, 2 patients (Pt09 and Pt23) had grade 1 neurologic adverse events in the humanized group and 2 patients (Pt11 and Pt14) developed grade 3 neurotoxicity in the murinized group. No abnormalities were detected in the CSF as assayed via lumbar puncture or in brain parenchyma by magnetic resonance imaging. Regardless of severity, the incidences of CRS and ICANS were similar between the two groups (Figure 6A,B).

The majority of severe adverse events (AEs) (grade 3 or higher) within the first month were cytopenia, including lymphopenia, neutropenia, thrombocytopenia, and anemia. Other treatment-related AEs were mainly low grade and reversible, including CRS, hypotension, hypoxia, heart failure, impaired liver function, lung infection, coagulopathy, diarrhea, metabolism and nutrition disorders, and effusion, and are detailed in Table 2. There were no fatal AEs (grade 5) and no treatment-related deaths.

AEs including CRS and ICANS were managed in accordance with protocol-specific guidelines as previously published. Most AE-associated symptoms including neurotoxicity were relieved or reversed after receiving nonsteroidal anti-inflammatory drugs (NSAIDs), tocilizumab, glucocorticoids, and valid symptomatic treatment.

### 3.6. High-Grade CRS Was Associated with Higher Levels of Cytokines in Vivo

**Figure 7:**
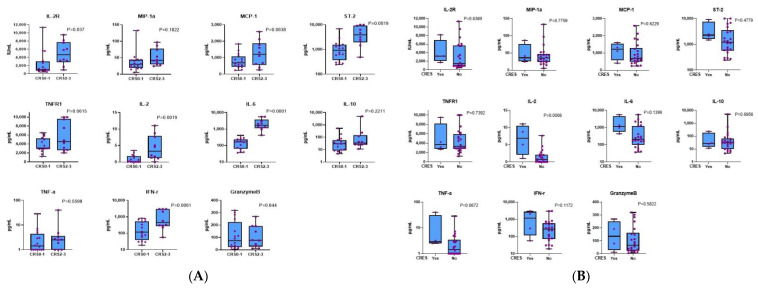
Peak values of cytokines in subgroups of patients. (**A**) Patients with grade 2–3 CRS had significantly higher peak levels of cytokines, including IL-6, IL-2, IL-2R, ST-2, and IFN-γ, than those with grade 1 CRS or without CRS. (**B**) The levels of the majority of cytokines had no significant association with ICANS grade, except IL-2. CRS: cytokine release syndrome; ICANS: immune effector cell-associated neurotoxicity syndrome.

**Figure 8:**
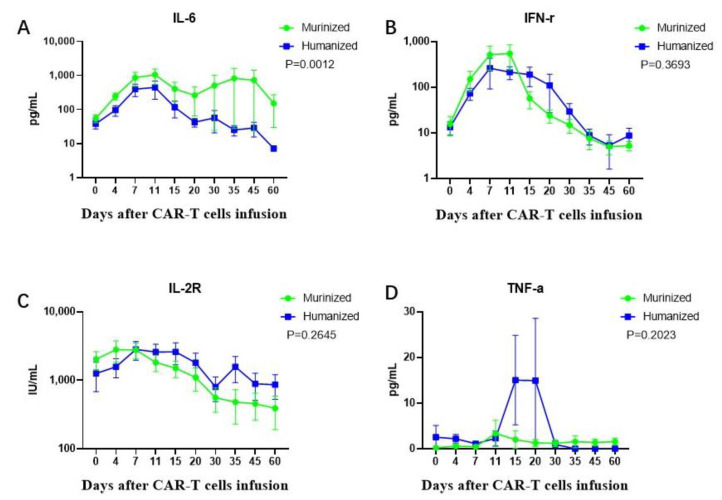
Comparison of cytokine levels in the murinized and humanized groups. Post CAR-T infusion, The murinized group had higher levels of IL-6 (**A**) than the humanized group (*p* = 0.0012). The trend of levels of IFN-r (**B**), IL-2R (**C**) and TNF-a (**D**) were similar in the two groups. TNF-a: tumor necrosis factor alpha; IFN-r: interferon gamma.

A panel of cytokines associated with CAR T-cell proliferation previously reported were assessed using patient serum samples before and after cell infusion. Patients with grade 2–3 CRS had significantly higher peak serum levels of inflammatory factors, including IL-6, IL-2, IL-2R, ST-2, IFN-γ (Figure 7A), than those with grade 1 CRS or without CRS. A similar association was observed between the occurrence of ICANS and the peak concentrations of IL-2 and TNF-α (Figure 7B). Levels of cytokines were not associated with CAR T-cell doses in either cohort. There was a similar trend in the levels of most cytokines, such as IFN-r, IL-2R, and TNF-a between the murine and humanized groups, except for IL-6, which was significantly different between the two groups (*p* = 0.0012) (Figure 8). Despite TNF-a secreted by CAR-T cells suddenly upregulated on day 11 after CAR-T infusion in the humanized group, this cytokine remained at relatively low levels insufficient to cause a severe CRS.

### 3.7. Humanized CAR T-Cells Proliferated More and Persisted Longer Compared to Murinized CAR T-Cells

**Figure 9:**
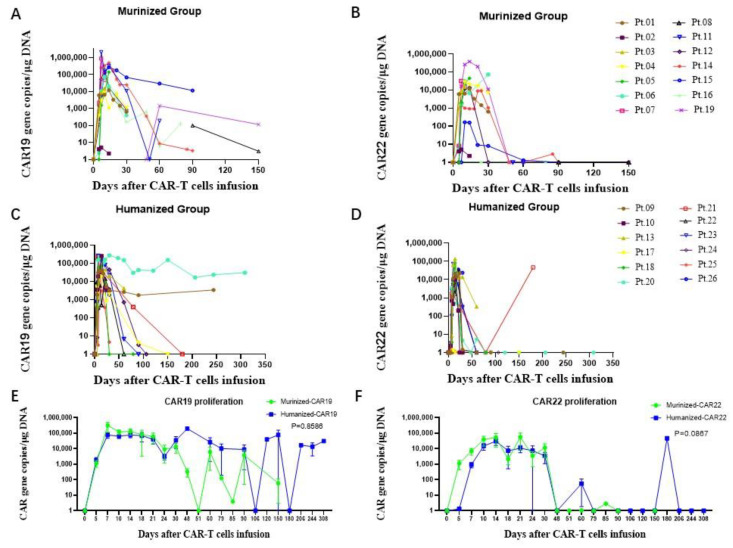
CAR-T cell proliferation in PB measured by qPCR. In the murinized group, (**A**) the median peak of circulating CD19 CAR-T cells, detected by qPCR, was 106,000 copies/μg genomic DNA (range: 5.07–2,090,000), occurring on day 10 (range: day 7–day 60); (**B**) the median peak for CD22 CAR-T cells was 12,850 copies/μg genomic DNA (range: 1–328,000), occurring on day 14 (range: day 7–day 30). (**C**) In the humanized group, the median peak of circulating CD19 CAR-T cells was 122,500 copies/μg genomic DNA (range: 18,600–280,000), occurring on day 12 (range: day 7–day 30); (**D**) the median peak of circulating CD22 CAR-T cells was 27,150 copies/μg genomic DNA (range: 1.17–136,000), occurring on day 14 (range: day 7–day 180). The comparison of the proliferation of the CAR19 cells (**E**) and CAR22 cells (**F**) in the murinized and humanized group. There was no significant difference between the CAR-T cell proliferation between the two groups, but the duration of the humanized CAR-T cells was longer than that of the murinized CAR-T cells. PB: peripheral blood; CAR-T: chimeric antigen receptor-T cell; qPCR: quantitative real-time polymerase chain reaction.

**Figure 10:**
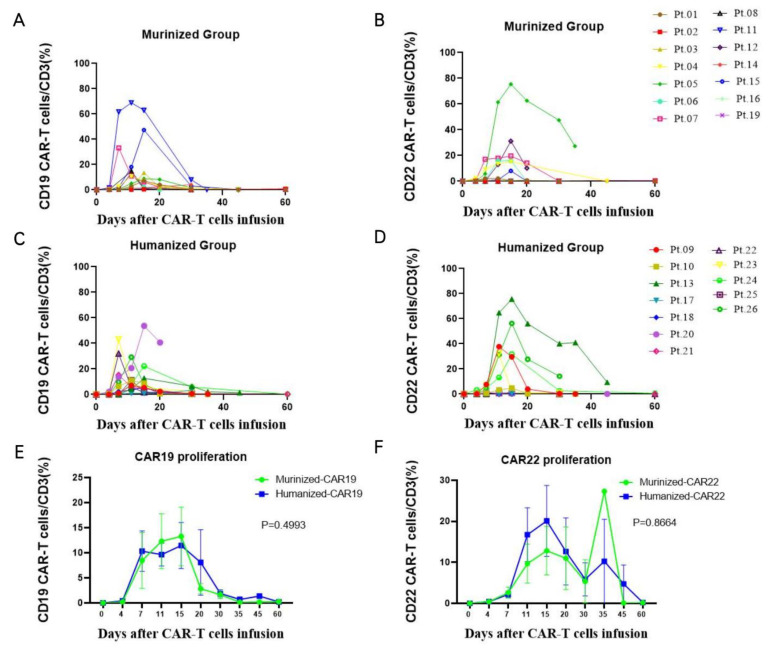
CAR-T cell proliferation in PB measured by FCM. In the murinized group, (**A**) the peak of CD19 CAR-T cells was 9.84% (range: 0.06%–68.93%) and occurred on day 15 (range: day 7–day 60), (**B**) and the peak of CD22 CAR-T cells was 1.96% (range: 0.04–75.44%) and occurred on day 13 (range: day 4–day 30). In the humanized group, the peak of CD19 CAR-T cells (**C**) and CD22 CAR-T cells (**D**) was 14% (range: 3.28–53.74%) on day 11 (range: day 7–day 30) and 3.03% (range: 0.09–75.7%) on day 15 (range: day 4–day 20), respectively. There were no significant differences in the CAR19 cell (**E**) and CAR22 cell proliferation (**F**) between the two groups. FCM: flow cytometry; PB: peripheral blood; CAR-T: chimeric antigen receptor-T cell.

**Figure 11:**
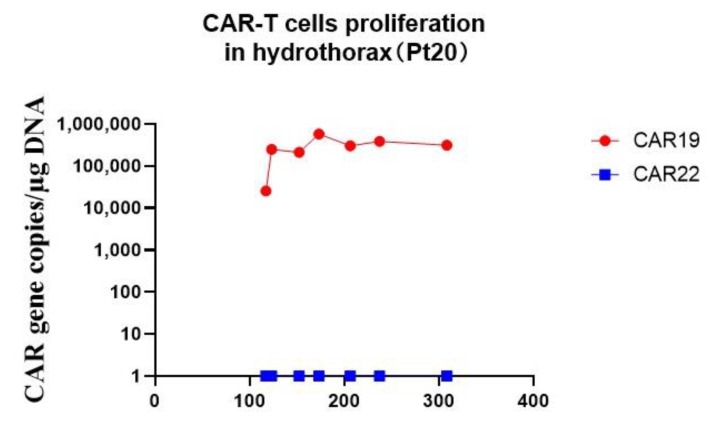
CAR-T cell proliferation in the hydrothorax of Pt20. High and persistent proliferation of CD19 CAR-T cells in the hydrothorax of Pt20 following CAR-T infusion. The CAR19 cells were still detectable one year after infusion.

The CD19 and CD22 CAR T-cells in the peripheral blood were detected by both qPCR and flow cytometry following cell infusion (Figure 9 and Figure 10). In the murinized group, the median peak number of circulating CD19 CAR T-cells was 106,000 copies/μg genomic DNA (range: 5.07–2,090,000), reached around day 10 (range: day 7–day 60) and the CD22 CAR T-cells was 12850 copies/μg genomic DNA (range: 1–328,000), occurring around day 14 (range: day 7–day30) (Figure 9A,B). In the humanized group, the median peak number of circulating CD19 CAR T-cells was 122,500 copies/μg genomic DNA (range: 18,600–280,000), which was achieved around day 12 (range: day7–day 30), and the CD22 CAR T-cells was 27,150 copies/μg genomic DNA (range: 1.17–136,000), which occurred around day 14 (range: day 7–day 180) (Figure 9C,D). Peak expansion did not significantly differ between murinized group and humanized group or dose level, while the duration of CAR T-cells in the humanized group was longer than in the murinized group (Figure 9E,F). Furthermore, we observed high and persistent presence of CD19 CAR T-cells beyond one year in the hydrothorax of patient 20. However, genomic DNA replications of CD22 CAR T-cells were not detected in the hydrothorax (Figure 11).

The CD19 CAR T-cell proliferation reached a level as high as 68.9% of total T-cells as determined by FCM, with a median peak proportion of 9.84% (range: 0.06–68.93%) and a median peak time on day 15 (range: day 7–day 60). The median peak proportion of CD22 CAR-T was 1.96% (0.04–75.44%), occurring on day 13 (range: day 4–day 30) in the murinized group (Figure 10A,B). In the humanized group, the median peak proportion of CD19 CAR-T cells and CD22 CAR-T was 14% (range: 3.28–53.74%) on day 11 (day 7–day 30) and 3.025% (range: 0.09–75.68%) on day 15 (range: day 4–day 20), respectively (Figure 10C,D). Cerebrospinal fluid was extracted from 10 patients though lumbar puncture, including 6 cases in the murinized group and 4 cases in the humanized group. The median peak proportion of CD19 CAR-T was 5.6% (range: 0.01–35.5%) on day 15, and the median peak proportion of CD22 CAR-T was 2.03% (range: 0.01–27.3%), occurring on day 15. As with the level of amplified genomic DNA detected by qPCR, there was no significant difference in the peak proportion in PB and CSF between the two groups.

**Figure 12:**
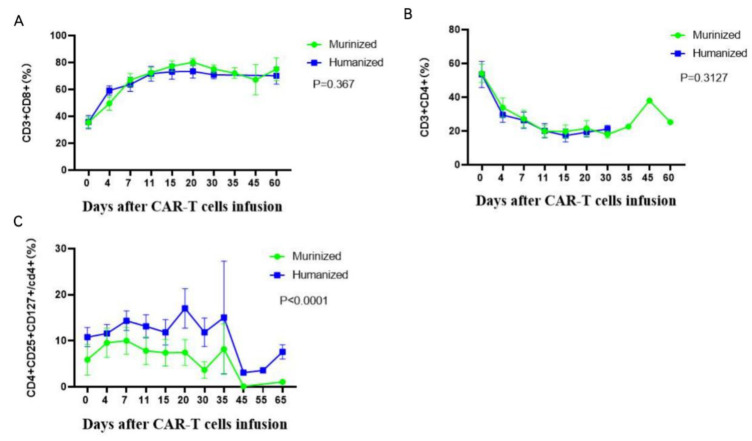
Comparison of T lymphocyte subsets in the murinized and humanized groups. Similar percentage of CD3^+^CD8^+^ (**A**) and CD3^+^CD4^+^ (**B**) in the two groups, while the percentage of CD4^+^CD25^+^CD127^LOW+^ cells (**C**) in the humanized group was much higher than in the murinized group (*p* < 0.0001).

Changes in T lymphocyte subsets in PB, detected by flow cytometry post infusion, were consistent with the CAR T-cell proliferation. CD3^+^ CD8^+^ cytotoxic-cells rapidly proliferated to peak levels on day 15 and then maintained or decreased slightly by day 60, accompanied by a gradual decline of CD3^+^ CD4^+^ helper T-cells in peripheral blood (Figure 12A,B). There was a similar trend for CD3^+^CD4^+^ and CD3^+^CD8^+^ T-cells in the murinized and humanized groups. We detected a significant difference in the CD4^+^CD25^+^CD127^low^ regulatory T-cells between the two groups (*p* < 0.0001) (Figure 12C).

## 4. Discussion

Clinical data continue to demonstrate that CD19 CAR T-cell therapy is effective for patients with certain R/R B-cell hematological malignancies. However, insufficient persistence and targeted antigen loss have emerged as major challenges for long-term survival following CAR T-cell therapy, especially in patients with high-risk features [6,11,24,25,26,27,28]. Therefore, the strategy of a fully humanized scFv and dual-target CAR T-cells has potential to be a treatment option for those patients with an unfavorable prognosis, including those that have relapsed after initial murinized CAR T-cell therapy [29,30]. Our current study has demonstrated promising clinical efficacy and manageable toxicity of a mixed infusion of CAR19/22 T-cell cocktail in patients with R/R B-NHL. In parallel, we also compared efficacy and safety results among two groups of patients that received either a humanized or a murinized version of this cellular product.

Based on the previously published ZUMA-1, JULIET, and TRANSCEND trials, first-in-class CD19 CAR-T cell products have been approved by the U.S. Food and Drug Administration (FDA). These studies showed unprecedented ORRs of 41% to 83% and approximately 50% CR rates among chemo-refractory aggressive B-NHL patients [2,31,32,33]. Robust responses were also achieved in our study, whether in the humanized or murinized group. On day 28 post-infusion, we achieved an ORR of 91.7% (11/12) and a CR rate of 75% (9/12) in the humanized group in our trial, and a similar ORR of 92.9% (13/14) and a CR rate of 42.9% (6/14) in the murinized group. Particularly, 75% of patients (9/12) with CR maintained their responses, achieving long-term survival in the humanized group at the 3-month assessment. Similarly, at a median follow-up of 11 months, the median PFS of the murinized group was 5 months, in line with the 6-month PFS reported in the ZUMA-1 series trials. The median PFS of our humanized group has not yet been reached, suggesting an advantage in long-term prognosis in this patient group.

Furthermore, most of the enrolled relapsed or refractory B-NHL patients were characterized by high risk factors, such as a history of prior CAR-T exposure, highly aggressive double-hit or Burkitt lymphoma, extremely high tumor burden with bulky mass, and exceedingly adverse genomic aberrations, particularly in the humanized group. Features were kept as balanced as possible between the two groups, with the exception of the 4 patients who failed previous murinized CD19 CAR-T therapy and received humanized CAR-T products in our study, and 2 patients who achieved CR and have maintained CR up to the data cut-off. More importantly, the dose of CD22 CAR-T cells infused in the humanized group with higher efficiency was significantly lower than that in the murinized group, as the first few patients who received the lowest dose of humanized CAR T-cell cocktail therapy achieved satisfactory efficacy, and one case of grade 3 CRS. Therefore, there were no further dose escalations performed. Fully humanized or dual-target CAR T-cells may be an alternative treatment for patients who have relapsed after a prior murinized or single-target CAR T-cell therapy. It is encouraging that 4 Burkitt lymphoma patients also achieved a best ORR of 100%, and only 1 patient relapsed early and died of rapid disease progression, while the other 3 patients achieved long-term survival, including a patient who bridged to allo-HSCT.

The TP53 gene is called ‘the guardian of the genome’ and represents a key tumor suppressor gene regulating DNA repair pathways, apoptotic death, and autophagy [34,35]. Mutations of the TP53 gene remain clonally stable even following multiple lines of therapies, which predicts a high probability of primary chemoresistance, early relapse or progression, and extremely poor prognosis [36]. In our previous study analyzing 254 B-ALL patients, compared to the TP53 wild-type group, patients with TP53 mutations had lower CR rates, resulting in significantly poorer OS and LFS, despite receiving CD19 CAR-T cell therapy [37]. In a multicenter, randomized, phase 3 trial, 5-year OS rates were 81% and 33% in wild type and TP53 mutation type cases, respectively, and the 5-year PFS rates were 73% and 19% respectively [38,39]. The standard salvage chemotherapy with subsequent HDT/ASCT still cannot overcome the poor prognosis conferred by the presence of a TP53 mutation. Studies have shown that dual-targeted CAR T-cell therapy offers a potential solution to improve patient prognosis in those patients with TP53-mutation. Huang et al. reported an ORR and CR rate of 87.1% and 45.2%, respectively, among patients with TP53 alterations treated with CAR19/22 T-cell cocktail therapy [40]. Furthermore, we observed that the patients in the humanized group who received CAR19/22 T-cell cocktail therapy had better efficacy outcomes compared to the patients treated with the murinized CAR T-cell therapy. Of five patients with TP53-positive disease who received humanized CAR19/22 T-cell cocktail therapy, 4 achieved long-term remission without relapse, likely due to the long-term persistence of humanized CAR-T cells in vivo.

In the humanized CAR-T group, CAR19 or CAR22 T-cells could still be detected in the PB and CSF samples months to one year later. Interestingly, CAR T-cells were also detected abundantly in the hydrothorax. High levels of CD19 CAR T-cells were shown to persist for more than one year in the hydrothorax of Pt20 after receiving humanized CAR T-cells, which may have the potential to mediate long-term disease remission in this patient. Therefore, more efforts should be made to prolong the persistence of CAR T-cells in vivo to extend the duration of remission.

Similar to regulatory T-cells (Tregs), CD4^+^CD25^+^CD127^low^ T-cells were often considered immunosuppressive, and were associated with poor prognosis following CD19 CAR-T therapy for R/R B-ALL [41]. However, Tregs also play essential roles in maintaining immunological self-tolerance and preventing autoimmunity such as graft-versus-host disease (GVHD). Adoptive infusion of CAR-Tregs are even explored in clinical trials as a novel approach to treat autoantibody-mediated autoimmune diseases [42,43]. Interestingly, we found that the levels of CD4^+^CD25^+^CD127^low^ T-cells in the humanized CAR-T group were significantly higher than that in the murinized group, especially at 15–30 days after the infusion when the CAR-T cell levels gradually declined from peak values. Therefore, we boldly speculate that the phenomenon may be related to the immunogenicity of CAR-T and the auto anti-CAR immune response limiting the persistence of CAR T-cells. Testing this hypothesis will require larger patient trials and further basic research.

Importantly, the CAR19/CAR22 T-cell cocktail regimen was generally well tolerated with moderate toxicity in the patients in our trial. The severe AEs were mostly cytopenia, and no patient had a treatment-related fatal AEs. Nearly all patients experienced CRS, but the vast majority were mild (grade 1–2) and reversible, and only 2 patients developed grade 3 CRS, consistent with a previously reported sequential infusion of CAR19/CAR22 T-cell cocktail therapy [29]. Thus, the mixed infusion of the CAR19/22 T-cell cocktail was also an efficient and well-tolerated approach. It is worth noting that 2 cases of grade 3 ICANS occurred in the murinized group, while only 2 cases of grade 1 ICANS occurred in the humanized group. The low rate of toxicity may be related to the low levels of induced inflammatory cytokines. The levels of inflammatory cytokines were similar to the observed basal level, or with only mild increases.

In conclusion, this clinical trial demonstrated promising efficacy and safety of CD19/CD22 CAR-T cocktail therapy for R/R aggressive B-cell lymphoma. Although this study was not a randomized trial, in our parallel comparison, patients who received humanized CAR T-cells achieved a higher CR rate than those treated with the murinized CAR-T. Patients in the humanized group also had a better PFS than those in the murinized group, particularly those harboring a TP53 mutation. Longer-term observation of these patients and larger randomized controlled studies of this type of dual-targeted CAR-T cell therapy approach are needed.

## Figures and Tables

**Figure 3:**
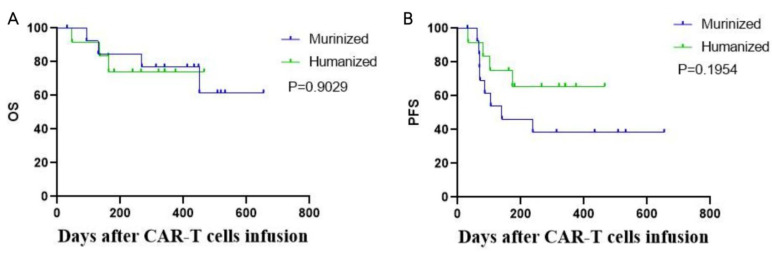
Survival curve comparison between the murinized and humanized groups. (**A**) No difference in overall survival between the murinized and humanized groups; median OS not achieved in either group. (**B**) Trend towards better PFS in the humanized compared to the murinized group. (*p* = 0.1954). OS: overall survival; PFS: progression-free survival.

**Table 1:** Patients’ baseline characteristics.

Characters	Murinized Group (*n* = 14) No. (%)	Humanized Group (*n* = 12) No. (%)
**Diagnosis**		
DLBCL	10 (71.4)	10 (83.3)
BL	2 (14.3)	2 (16.7)
FL	2 (14.3)	0 (0.0)
**Gender**		
Male	11 (78.6)	9 (75.0)
Female	3 (21.4)	3 (25.0)
**Median age years, (range)**	50.5 (29–66)	46 (4–75)
≤18	0 (0.0)	2 (16.7)
>18	14 (100.0)	10 (83.3)
**Prior lines of chemotherapy**		
≤2	6 (42.9)	3 (25.0)
>2	8 (57.1)	9 (75.0)
**History of prior radiotherapy**		
Yes	3 (21.4)	5 (41.7)
No	11 (78.6)	7 (58.3)
**History of prior CD19 CAR-T**		
Yes	0 (0.0)	4 (33.3)
No	14 (100.0)	8 (66.7)
**IPI**		
2	4 (28.6)	0 (0.0)
3	4 (28.6)	9 (75.0)
4 or 5	6 (42.8)	3 (25.0)
**Transplant Status**		
Relapsed from transplant	1 (7.1)	1 (8.3)
No previous transplant	13 (92.9)	11 (91.7)
**High-risk factors**		
Double hit	3 (21.4)	4 (33.3)
TP53 mutation	6 (42.9)	5 (41.7)
Bulky disease (≥7.5 cm)	9 (64.3)	2 (16.7)

Abbreviations: DLBCL: diffuse large B-cell lymphoma; BL: Burkitt lymphoma; FL: follicular lymphoma; IPI: International Prognosis Index.

**Table 2:** CRS, ICANS, and adverse events post CAR-T cells infusion.

	Murinized Group (*n* = 14), No. of Patients (%)			Humanized Group (*n* = 12), No. of Patients (%)		
Type of Event	Any Grade	Grade 3	Grade 4	Any Grade	Grade 3	Grade 4
**CRS**	13 (92.8)	1 (7.1)	0 (0.0)	12 (100)	1 (8.3)	0 (0.0)
**ICANS**	2 (14.3)	2 (14.3)	0 (0.0)	2 (16.7)	0 (0.0)	0 (0.0)
**Detail Adverse Events**						
**Renal disorder**	2 (14.3)	0 (0.0)	0 (0.0)	2 (16.7)	0 (0.0)	0 (0.0)
**Coagulopathy**	10 (71.4)	2 (14.3)	0 (0.0)	9 (75.0)	1 (8.3)	0 (0.0)
**Prothrombin time prolonged**	10 (71.4)	0 (0.0)	0 (0.0)	9 (75.0)	1 (8.3)	0 (0.0)
**Activated partial thromboplastin time prolonged**	1 (7.1)	0 (0.0)	0 (0.0)	4 (33.3)	1 (8.3)	0 (0.0)
**Fibrinogen decreased**	5 (35.7)	1 (7.1)	0 (0.0)	8 (66.7)	0 (0.0)	0 (0.0)
**General conditions**						
Hypertension	8 (57.1)	0 (0.0)	0 (0.0)	7 (58.3)	0 (0.0)	0 (0.0)
Hypotension	4 (28.6)	0 (0.0)	0 (0.0)	2 (16.7)	0 (0.0)	0 (0.0)
Hypoxia	9 (64.3)	0 (0.0)	0 (0.0)	8 (66.7)	0 (0.0)	0 (0.0)
**Laboratory values**						
AST increase	9 (64.3)	0 (0.0)	0 (0.0)	8 (66.7)	3 (25)	0 (0.0)
ALT increase	9 (64.3)	0 (0.0)	0 (0.0)	11 (91.7)	2 (16.7)	0 (0.0)
**Hematologic event**						
Myelosuppression	14 (100)	0 (0.0)	14 (100)	12 (100)	0 (0.0)	12 (100)
Neutropenia	14 (100)	2 (14.3)	12 (85.7)	12 (100)	1 (8.3)	11 (91.7)
Lymphopenia	14 (100)	0 (0.0)	14 (100)	12 (100)	0 (0.0)	12 (100)
Thrombocytopenia	14 (100)	6 (42.9)	8 (57.1)	12 (100)	6 (50.0)	6 (50.0)
Anemia	14 (100)	8 (57.1)	3 (21.4)	12 (100)	8 (66.7)	3 (25)
**Gastrointestinal event**						
Diarrhea	5 (35.7)	0 (0.0)	0 (0.0)	9 (75.0)	0 (0.0)	0 (0.0)
**Cardiovascular event**						
Heart failure	8 (57.1)	0 (0.0)	0 (0.0)	7 (58.3)	1 (8.3)	0 (0.0)
**Infection**						
Lung infection	4 (28.6)	0 (0.0)	0 (0.0)	7 (58.3)	1 (8.3)	0 (0.0)
**Metabolism and nutrition disorders**						
Hyponatremia	7 (50.0)	0 (0.0)	0 (0.0)	12 (100)	0 (0.0)	0 (0.0)
Hypokalemia	9 (64.3)	0 (0.0)	0 (0.0)	11 (91.7)	0 (0.0)	0 (0.0)
Hypocalcemia	11 (78.6)	0 (0.0)	0 (0.0)	12 (100)	0 (0.0)	0 (0.0)

Abbreviations: CRS: cytokine release syndrome; ICANS: immune effector cell-associated neurotoxicity syndrome; AST: aspartate aminotransferase; ALT: alanine aminotransferase.

## Data Availability

The data are not publicly available due to privacy or ethical restrictions. The data that support the findings of this study are available on request from the corresponding author.

## Supplementary Materials

The following supporting information can be downloaded at: https://www.mdpi.com/article/10.3390/cells11244085/s1, File S1: Eligibility Criteri; File S2: Cell production; File S3: qPCR to quantitate blood CAR-T cells; File S4 Flow cytometry; Table S1: Patient characteristics; Figure S1: Construct design of CD19 and CD22 CAR-T cell cocktail; Figure S2: The mean fluorescence intensity (MFI) of CAR expression.
