# Supplementary material for: Safety and Efficacy of Humanized Versus Murinized CD19 and CD22 CAR T-Cell Cocktail Therapy for Refractory/Relapsed B-Cell Lymphoma
Source: Cells. 2022 Dec 16;11(24):4085. doi: 10.3390/cells11244085 (PMC9776474; doi:10.3390/cells11244085)
Supplement: Supplementary file 1 [file cells-11-04085-s001.zip › cells-2024498-supplementary.pdf]

Supplementary methods

S1 Eligibility Criteria

S2 Cell production

S3 qPCR to quantitate blood CAR-T cells

S4 Flow cytometry

Supplementary Tables

Supplementary Table S1. Patient characteristics

Supplementary Figures

Supplementary Figure S1. Construct design of CD19 and CD22 CAR-T cell cocktail

Supplementary Figure S2. The mean fluorescence intensity (MFI) of CAR expression

Supplementary methods

S1. Study inclusion and exclusion criteria

Eligibility Criteria

Inclusion criteria

1) Sign the informed consent form, and willing and able to comply with the visits, treatment plans, laboratory tests, and other requirements of the study specified in the trial procedure table

2) Patients with confirmed relapsed or refractory non-Hodgkin lymphoma, definition of relapsed or refractory: Histo-pathologically diagnosed DLBCL, PMBCL, or TFL patients with primary resistance to standard treatment regimens; or PD developed after standard treatment with at least one second-line standard treatment regimen; or last treatment effect was SD and the duration did not exceed 6 months; or CD20 positive patients who did not respond to or relapsed after anti-CD20 monoclonal antibody treatment; or PD developed after autologous hematopoietic stem cell transplantation; or biopsy-proven relapse within 12 months; or patients who received salvage therapy after autologous hematopoietic stem cell transplantation who did not respond to last-line therapy or relapsed after therapy;

3) According to RECIST version 1.1, at least one measurable tumor focus;

4) Eastern Cooperative Oncology Group activity status score (Eastern Cooperative Oncology Group, ECOG)$\leqslant$3 points;

5) Age 3-75 years old, male or female

6) Tumor cells positive for CD19 and/or CD22 by immunohistochemistry or flow cytometry;

7) The expected survival period of greater than 3 months;

8) Main organ function meets the following conditions: serum creatinine $\leqslant$ 1.5 times the upper limit of normal (ULN); ALT$\leqslant$2.5 ULN; AST$\leqslant$2.5 ULN;

total bilirubin≤1.5 ULN; left ventricular ejection fraction (LVEF)≥ 45%; hemoglobin≥90g/L; platelet count≥50×10^9/L; absolute neutrophil count (ANC) ≥1.0×10^9/L; blood oxygen saturation>90%;

9) Collection time of patient's peripheral blood mononuclear immune cells must be at least 2 weeks from the latest radiotherapy of the patient or systemic therapy

Exclusion Criteria

1) Severe cardiac insufficiency;

2) History of severe pulmonary dysfunction;

3) History of other malignant tumors, except for non-melanoma skin cancer or carcinoma in situ (such as cervical cancer, bladder cancer, breast cancer) who have received radical treatment and no disease recurrence at least 2 years before screening;

4) Has severe infection or persistent infection that cannot be effectively controlled;

5) Has a metabolic disease (except diabetes and dyslipidemia);

6) Has severe autoimmune disease or congenital immunodeficiency;

7) Has active hepatitis (positive for hepatitis B virus deoxyribonucleic acid [HBVDNA] or hepatitis C virus ribonucleic acid [HCVRNA]);

8) Human immunodeficiency virus (HIV) infection or syphilis infection;

9) Has a history of severe allergy to biological products (including antibiotics);

10) In the past three months, participated in any other clinical drug trials (except CAR-T product clinical trials);

11) For female patients, pregnant and/or breastfeeding, or planning to become pregnant within 12 months;

12) Conditions that the investigator deems may increase the risk of the subject or interfere with the test results (with a history of serious mental illness, drug abuse and addiction, etc.).

S2. CAR-T cell manufacture

The CD19 and CD22 dual CAR-Tcells were manufactured in a cGMP facility. The structure of the murinized CD19 CAR consists of an anti-CD19 scFv (FMC63 murine monoclonal antibody), a CD8 hinge and transmembrane domain, 2 costimulatory domains from CD28 and 4-1BB, and an CD3 ζ intracellular domain. CD19 CAR encodes a truncated EGFR sequence, and the CD22 CAR encodes an anti-PDL1 ScFv.

The manufacturing process of CAR-T cells includes T- cell activation, transfection, expansion and harvesting. In brief, peripheral lymphocytes were collected from each patient by lymphopheresis and further separated by density gradient centrifugation on a Ficoll-Paque centrifuge (25910, Dongfang Huahui, Beijing). Then, T-cells were isolated from PBMCs using CD3+ microbeads (130-097-043, Miltenyi Biotec, Germany), followed by PBS supplemented with 2mM l-glutamine (Gibco), 5% human AB serum and 200IU/ml rhIL-2 (PeproTech), TexMACS GMP medium (170-076-306, Miltenyi Biotec, Germany) and stimulated with Dynabead™ Human T-Activator CD3/CD28 (Invitrogen) at a 1:1 ratio and incubated for 2 days. T-cells were then individually transduced with CAR19 lentivirus in the presence of 8 ug/ml protamine sulfate (H11020247, Yuekang Kaiyue, Beijing). The transduction rate of CAR-T cells was measured by flow cytometry on day 5, following three days of transduction. CAR-T cells continued to expand in TexMACS medium supplemented with 200 IU IL-2 until sufficient cell product was obtained on days 12-14. The humanized CAR-T cells were developed by replacing the entire mouse-derived scFv with fully human scFvs based on the backbone of a murine-based CAR to weaken their immunogenicity. CAR-T cells were manufactured in the presence of saline supplemented with 2% HSA. Quality control procedures included determination of transfection efficiency, apoptosis, tumoricidal activity, sterility, mycoplasma contamination, and endotoxin levels. All CAR-T cell products met specified specifications.

S3. qPCR to quantitate blood CAR-T cells

PBMCs were collected from each patient at multiple time points before and after treatment, and DNA was extracted from the PBMCs using the Qiagen DNeasy's kit. DNA at each time point was amplified using primer and probe sets specific for the CAR (Applied Biosystems). Real-time PCR was performed using the Roche Light Cycler 480 Real-Time PCR System. The primer/probe sequences are: (sense primer: 5'- GAAAGCTGACTGCCCCTATTTG-3', antisense primer: 5'-GAGAGGAAGTGCTGGGAACAAT-3'). An 8-point standard curve was generated, each data point (sample, standard curve, and reference sample) was evaluated in triplicate and the mean value reported. To control the amount of DNA interrogated, 12-20 ng of input genomic DNA was used for parallel amplification reactions and human genomic DNA reference assays with primer/probe combinations specific for the non-transcribed genomic sequence upstream of the CDKN1A gene. The amplification A correction factor (CF) was generated for the augmented response. Transgene copy number was calculated per microgram of DNA according to the following formula: copy number calculated from the standard curve/input DNA (ng) $\times$ CF $\times$ 1000 ng. The accuracy of this assay depends on the ability to quantify the labeling of the injected cellular product by qPCR.

S4. Flow cytometry

Multiparametric immunophenotyping assessment of human PBMCs by flow cytometry (BD FACSAria, USA) after Ficoll-Paque treatment using approximately 4×10^6 total cells/condition using fluorescence minus one (FMO) staining. For CSF samples, cells were collected after centrifugation. Cells were stained and incubated with the corresponding antibody master mix for 20 min at room temperature, then washed twice with flow buffer. Cells were then resuspended in PBS containing 0.5% paraformaldehyde to obtain approximately 2-3 million cells per staining mixture. Compensation values were established using single antibody staining and BD compensation beads (Becton Dickinson) and were automatically calculated and applied by the instrument software. Data were analyzed using FlowJo software.

Supplementary Table 1. Patient characteristics at baseline and responses post CAR-T cell infusion

Murinized Group

Patient No.	Age (years)	Gender	Diagnosis	History of prior Auto-HSCT	History of prior CD19 CAR-T	CD19 dose infused ($\times 10^6$/kg)	CD22 dose infused ($\times 10^6$/kg)	Efficacy evaluation on Day 28	Efficacy evaluation on Month 3	CRS Grade	ICANS Grade	Hormone use	Tocilizumab use
Pt.01	29	M	BL	No	No	2	1	CR	PD	1	0	No	No
Pt.02	57	M	DLBCL	No	No	1	1	PR	PR	1	0	No	No
Pt.03	58	M	DLBCL	No	No	1	1	SD	/	0	0	No	No
Pt.04	57	M	DLBCL	No	No	3	3	PR	PD	2	0	No	No
Pt.05	41	F	DLBCL	No	No	3	1	CR	CR	1	0	Yes	No
Pt.06	38	M	DLBCL	No	No	3	3	PR	PR	1	0	No	No
Pt.07	45	M	DLBCL	Yes	No	3	3	CR	CR	1	0	No	No
Pt.08	30	M	BL	No	No	2	2	CR	CR	1	0	No	No
Pt.11	56	M	DLBCL	No	No	2	1	CR	CR	2	3	No	No
Pt.12	35	M	FL	No	No	3	3	PR	PR	1	0	Yes	Yes
Pt.14	65	M	DLBCL	No	No	2	2	PR	PD	3	3	Yes	Yes
Pt.15	66	F	DLBCL	No	No	1.5	1.5	PR	PD	1	0	No	No
Pt.16	64	M	DLBCL	No	No	2	1	PR	PD	1	0	No	No
Pt.19	33	F	FL	No	No	2	1	CR	CR	1	0	No	Yes

Humanized Group

Patient No.	Age (years)	Gender	Diagnosis	History of prior Auto-HSCT	History of prior CD19 CAR-T	CD19 dose infused ($\times10^6$/kg)	CD22 dose infused ($\times10^6$/kg)	Efficacy evaluation on Day 28	Efficacy evaluation on Month 3	CRS Grade	ICANS Grade	Hormone use	Tocilizumab use
Pt.09	61	F	DLBCL	No	Yes	2	0.5	CR	CR	2	1	No	No
Pt.10	47	M	DLBCL	No	Yes	3	3	SD	PD	2	0	No	No
Pt.13	45	M	DLBCL	No	Yes	2	0.5	PR	PD	2	0	Yes	Yes
Pt.17	58	M	DLBCL	No	Yes	1	0.5	CR	CR	1	0	Yes	Yes
Pt.18	17	M	BL	No	No	2	0.5	CR	CR	1	0	No	No
Pt.20	75	M	DLBCL	No	No	2	0.5	CR	CR	3	0	Yes	No
Pt.21	31	F	DLBCL	Yes	No	2	0.5	CR	CR	1	0	Yes	Yes
Pt.22	32	M	DLBCL	No	No	2	0.5	CR	CR	2	0	No	Yes
Pt.23	58	M	DLBCL	No	No	2	0.5	CR	CR	2	1	No	No
Pt.24	66	M	DLBCL	No	No	2	0.5	PR	SD	2	0	Yes	No
Pt.25	4	M	BL	No	No	1	0.5	CR	CR	1	0	No	Yes
Pt.26	38	F	DLBCL	No	No	2	0.5	CR	CR	1	0	No	No

Abbreviations: F: female; M: male; DLBCL: diffuse large B-cell lymphoma; BL: burkitt lymphoma; FL: follicular lymphoma; HSCT: hematopoietic stem cell transplantation; CR: complete response; SD: stable disease; PR: partial response; PD: progressive disease; CRS: cytokine release syndrome; ICANS: immune effector cell-associated neurotoxicity syndrome.

Supplementary Figure S1. Construct design of CD19 and CD22 CAR-T cell cocktail

A. CAR(CD19)-tEGFR

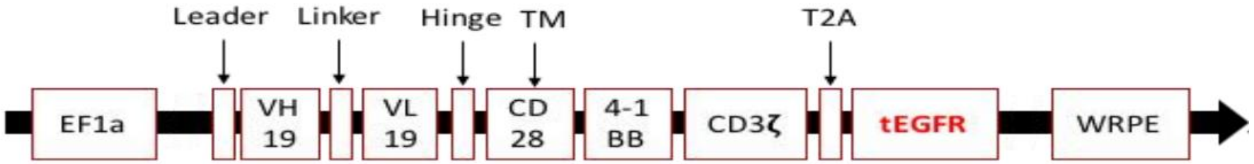

B. CAR(CD22)-aPDL1

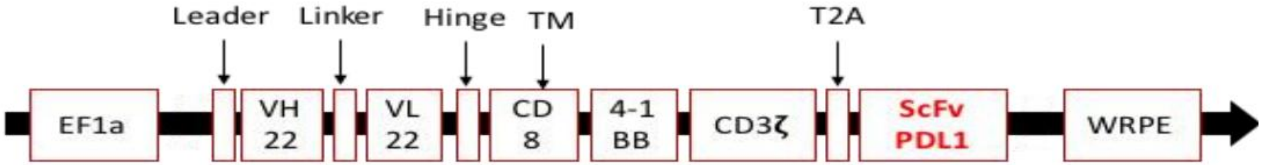

Supplementary Figure S2. The mean fluorescence intensity (MFI) of CAR expression

A. Mean fluorescence intensity (MFI) quantification of CD19 CAR-T cells staining with anti-EGFR antibody by flow cytometry.

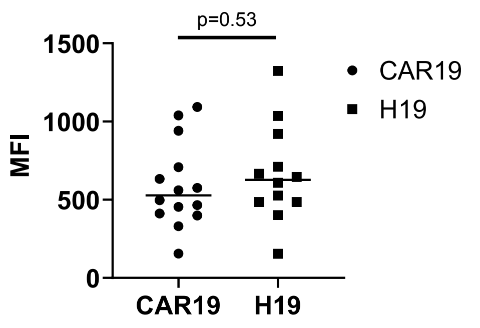

B. Mean fluorescence intensity (MFI) quantification of CAR22 T cells staining with CD22-Fc fused protein by flow cytometry.

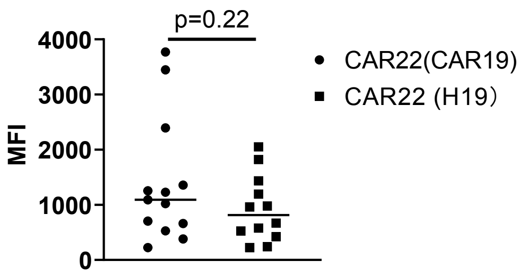

CAR19:Murinized group

H19:Humanized Group
